# Novel Therapeutic Strategies for Preventing Recurrent Urinary Tract Infections in Women: A Narrative Review

**DOI:** 10.3390/antibiotics15101030

**Published:** 2026-10-16

**Authors:** Dominik Zawadzki, Natalia Libergal, Mikołaj Kisiała, Hanna Gwoździk, Oskar Ratajczyk, Mateusz Hejnowicz, Piotr Misiąg, Łukasz Nowak, Dawid Janczak, Wojciech Krajewski, Tomasz Szydełko, Bartosz Małkiewicz

**Affiliations:** Department of Minimally Invasive and Robotic Urology, University Center of Excellence in Urology, Wroclaw Medical University, 50-556 Wroclaw, Poland; natalia.libergal@student.umw.edu.pl (N.L.); piotr.misiag@student.umw.edu.pl (P.M.); bartosz.malkiewicz@umw.edu.pl (B.M.)

**Keywords:** recurrent urinary tract infections in women, antibiotic prophylaxis, immunoprophylaxis

## Abstract

**Background**: It is estimated that approximately 20–30% of women suffer from recurrent urinary tract infections (rUTIs). The disease is primarily bacterial, especially *Escherichia coli*, making antibiotics the standard treatment. Increasing antibiotic resistance of uropathogens reduces treatment effectiveness. Therefore, non-antibiotic approaches are increasingly being investigated for the management of rUTIs. This narrative review provides a comprehensive overview of currently available and emerging methods of treatment and prevention of rUTIs in women. **Methods**: This paper employs a narrative review with a structured literature search and synthesizes data from three major databases, including PubMed, Web of Science, and Scopus. The review was conducted in accordance with the Scale for the Assessment of Narrative Review Articles (SANRA) checklist. Scientific articles were searched using the following keywords: ‘recurrent urinary tract infections in women’, ‘antibiotic prophylaxis’, ‘immunoprophylaxis’, ‘urinary microbiome’. **Results**: Observational evidence has reported an association between continuous antibiotic prophylaxis and severe outcomes, including pyelonephritis and septicemia; however, these findings are susceptible to confounding by indication and should not be interpreted as evidence of a causal relationship. Clinical studies suggest that immunomodulatory approaches, such as OM-89 bacterial lysate, next-generation polybacterial mucosal vaccines (e.g., MV140), and oral immunostimulants, have potential to reduce the frequency of rUTI recurrences in women, although the strength and certainty of evidence vary between interventions. Bacteriophage therapy is an emerging and exploratory targeted approach supported by early clinical and experimental evidence, but its efficacy for preventing recurrent uncomplicated UTIs remains uncertain, though rapid development of phage resistance remains a potential limitation. Prophylactic strategies, including *Lactobacillus*-based probiotics, low-dose estriol, and microbiota transplantation, have shown preliminary or variable results, with limited evidence for rUTI prevention. In particular, findings on vaginal dysbiosis and microbiota modulation should not be interpreted as a direct evidence of rUTI prevention. **Conclusions**: While antibiotic therapy remains essential, continuous prophylaxis may contribute to antimicrobial resistance and disrupt microbial balance. Therefore, novel therapeutic strategies are still being studied. Several non-antibiotic strategies, including immunoprophylaxis and microbiome modulation, represent potential preventive approaches, whereas bacteriophage therapy and vaginal microbiota transplantation remain exploratory and require further clinical validation. Their preventive efficacy against rUTIs remains unestablished. Moreover, antimicrobial peptides (AMPs), nanotechnology, molecularly targeted therapies, and precision medicine strategies are being investigated. Plant-based therapies may provide benefit, but their clinical efficacy remains to be established. Overall, further clinical studies are needed to confirm the preventive efficacy of these emerging approaches.

## 1. Introduction

Recurrent urinary tract infections (rUTIs) are a significant clinical and epidemiological problem in the female population worldwide. In women, rUTIs are commonly defined as ≥2 symptomatic episodes within 6 months or ≥3 episodes within 12 months. This definition is commonly applied to recurrent uncomplicated urinary tract infections, whereas these infections may also occur in the presence of complicating factors, including urinary catherization or structural and functional abnormalities of the urinary tract. The problem of recurrence stems from individual anatomical predispositions, functional factors, and hormonal disorders that promote colonization of the urinary tract by uropathogens. It is estimated that over 50% of women experience at least one episode of urinary tract infection (UTI) in their lifetime, while approximately 20–30% of women who have experienced an initial UTI develop a recurrent infection [1,2]. *Escherichia coli* remains the most commonly isolated bacterium, but other bacteria, including *Klebsiella pneumoniae*, *Staphylococcus saprophyticus*, and *Proteus mirabilis*, can also cause infections [2,3].

In addition to these common bacterial pathogens, certain viruses and protozoa can also contribute to urinary tract infections, especially in immunocompromised individuals. To date, antibiotics remain the mainstay of treatment for urinary tract infections, but their effectiveness is now limited by antibiotic-resistant bacterial strains. The resistance of uropathogens, particularly *E. coli*, to fluoroquinolones, trimethoprim-sulfamethoxazole, and other commonly used antibiotics significantly complicates empirical therapy [1,3]. Chronic and often excessive exposure to antimicrobial drugs is the primary driver of antibiotic resistance in urology [1,4].

There is also an increased risk of microbiota disorders and adverse effects associated with long-term treatment [1,4]. The growing resistance of multidrug-resistant strains makes urinary tract infections increasingly difficult to treat [3,4]. The problem of antibiotic resistance has increased the need to develop and implement non-antibiotic approaches [4,5,6]. Promising non-antibiotic therapies and complementary treatment strategies include immunoprophylaxis and microbiome modulation (including fecal microbiota transplantation [FMT], anti-adhesion therapies, and new molecules with distinct mechanisms of action) [5,6,7].

While recent reviews have addressed non-antibiotic prevention of rUTIs, this review additionally focuses on emerging therapeutic approaches and differentiates strategies according to the maturity of the available clinical and experimental evidence.

The aim of this paper is to provide a comprehensive overview of modern and future-oriented methods for the treatment and prevention of rUTIs in women, with particular emphasis on alternative, safe, non-antibiotic interventions [4,8].

## 2. Materials and Methods

This study was designed as a narrative review based on a structured literature search. The review was conducted in accordance with the Scale for the Assessment of Narrative Review Articles (SANRA) checklist to enhance transparency, methodological consistency, and reporting quality [9]. It should be noted that this review was not conducted as a systematic review, and no formal meta-analysis was applied.

Databases such as PubMed, Web of Science, and Scopus were used in the initial literature search. The search was performed from March 2026 to August 2026, with the final search conducted in August 2026. No predefined restriction on publication year was applied, and the search included relevant publications available up to August 2026. The literature search included combinations of the following key terms: “recurrent urinary tract infections in women”, “antibiotic prophylaxis”, “immunoprophylaxis”, and “urinary microbiome”, together with additional terms related to vaccines, probiotics, vaginal microbiota, microbiome modulation, bacteriophages, antimicrobial peptides, biofilms, and other emerging non-antibiotic strategies. The search strategy was iterative, allowing additional searches when specific topics or emerging preventive approaches required further clarification (Table 1).

The initial literature search and screening of titles and abstracts were performed by two authors. Duplicate records identified across the searched databases were removed before further assessment. Potentially relevant publications were subsequently assessed in full text, and the final selection was discussed among the authors based on relevance to the objectives and thematic scope of the review, study design, and availability of clinically relevant information. In cases of uncertainty regarding the relevance of a publication, the decision was discussed among the authors until consensus was reached.

At the full-text stage, publications were assessed for relevance to the review’s main objectives and thematic scope, as well as the availability of clinically relevant information. Studies addressing the same preventive strategies or clinical questions were considered based on the relevance and quality of the available evidence. Studies addressing catheter-associated biofilms, vaginal dysbiosis, or treatment of active urinary tract infections were included when they provided mechanistic or clinical evidence relevant to pathophysiology, microbial persistence, or potential prevention of rUTIs. Such studies were not considered direct evidence of efficacy in preventing rUTIs. This distinction was maintained throughout the evidence synthesis to avoid interpreting indirect, experimental, or preclinical findings as established clinical efficacy for the prevention of recurrent uncomplicated UTIs in women.

Studies were included if they were focused on preventive strategies for recurrent urinary tract infections in women, encompassing both well-established and novel therapeutic approaches. Conference abstracts, publications in languages not accessible to the authors, and publications lacking relevant data were excluded. No formal restriction based on publication date was applied.

Across the three databases, a total of 1535 records were identified. Following screening for relevance and removal of duplicate and irrelevant records, 98 publications were ultimately selected for inclusion in the narrative review. The final body of evidence included original clinical studies, observational studies, experimental studies, and relevant reviews, with the strength of evidence considered according to study design. Randomized clinical trials and well-designed clinical studies were given greater weight in the synthesis, whereas observational studies, uncontrolled cohorts, in vitro studies, and animal studies were interpreted according to their respective limitations and primarily used to provide supportive, mechanistic, or exploratory evidence. To facilitate a structured presentation of the evidence, the study’s literature was organized into the following sections: pathophysiology of recurrent urinary tract infections, limitations of conventional antibiotic therapy, immunoprophylaxis and vaccines, microbiome-based therapies, bacteriophage therapy, and other emerging approaches.

## 3. Pathophysiological Background of Recurrent UTIs

### 3.1. Intracellular Bacterial Communities and Biofilm Formation in UTI Persistence and Virulence

Biofilm formation, predominantly by *E. coli*, is common among catheterized patients [10]. *E. coli* generally forms weak biofilm [11], while uropathogenic *Escherichia coli* (UPEC) forms biofilms significantly more often than commensal strains [12]. In vitro biofilm formation is also more frequent among uropathogens isolated from women with recurrent UTIs than from those with isolated episodes [13]. Silicone catheters may reduce biofilm formation, whereas pharmacological approaches, such as JUC Spray Dressing, can further inhibit catheter-associated biofilms [14]. Importantly, biofilms may persist on urinary catheters despite antibiotic therapy, highlighting the need for novel antibiofilm strategies [15]. Experimental approaches, including chitosan-based rosemary oil nanophytosomes and azithromycin- and ciprofloxacin-impregnated urinary catheters, have demonstrated antibiofilm activity against uropathogens, including Pseudomonas aeruginosa [16,17]. Disperazol combined with ciprofloxacin has also shown promising results in a murine model of catheter-associated UTIs [18]. However, these studies predominantly address catheter-associated or experimental UTIs rather than the prevention of recurrent uncomplicated UTIs in women. Therefore, their findings provide mechanistic and proof-of-concept evidence rather than direct clinical evidence for rUTI prevention.

### 3.2. The Role of Adhesion

Adhesion is crucial for the invasion of urothelial cells in UTIs [19]. UPEC adhesion and invasion are associated with bladder inflammation [20], while adhesion-related virulence genes are more prevalent in the urinary tract than in fecal *E. coli* isolates [21]. Sheikhi et al. reported that biofilm-forming UPEC shows an increased prevalence of adhesin-associated genes, including fimH, kpsMTII, csgA, afa/draBC, and pap [22]. These findings support bacterial adhesion as a potential therapeutic and vaccine target. For example, immunization against *A. baumanii* adhesion-related antigens reduced bacterial loads in bladder tissue and on catheter surfaces [23]. FimH, a fimbrial-tip adhesin that binds mannosylated urothelial proteins, is another promising target, with FimH-based vaccination conferring protection against FimH-expressing *E. coli* and eliciting antibody responses associated with reduced bacterial binding [24]. Nevertheless, these findings are largely derived from experimental models and studies of bacterial virulence or active infection, and therefore should not be interpreted as direct evidence of efficacy in preventing recurrent uncomplicated UTIs in women. Although the aforementioned studies were conducted in a mouse model, they may provide valuable insights for the development of future therapies.

### 3.3. The Relation Between Vagina and Bowel Dysbiosis in UTIs

The intestinal microbiota is associated with susceptibility to UTIs, with recurrent UTIs patients exhibiting altered abundances of several taxa, including *Anaerotruncus*, *Dorea*, *Eubacterium nodatum*, *Odoribacter*, *Senegalimassilia*, Tyzzerella3, Lachnospiraceae ND3007 and UCG001 groups, Ruminococcaceae, and Bacillales [24]. Individuals with recurrent UTIs also exhibit reduced microbial richness and fewer butyrate-producing bacteria, suggesting a role for the gut–bladder axis in disease susceptibility and immune regulation [25]. These observations describe associations with dysbiosis and susceptibility rather than establishing a preventive effect against recurrent uncomplicated UTI. Gut dysbiosis may enhance inflammatory responses, impair the bladder epithelial barrier, and promote *E. coli* persistence. Fecal microbiota transplantation has therefore been proposed as a potential strategy to modulate excessive inflammation and reduce recurrent UTIs [26,27]. However, evidence for microbiota-based interventions remains preliminary, and these approaches should be distinguished from interventions with established clinical evidence for rUTI prevention. Similarly, vaginal *Lactobacillus* spp. inhibit UPEC growth through antimicrobial metabolites, thereby supporting a protective role of the vaginal microbiota [28]. Vaginal and combined oral-vaginal probiotics may reduce vaginal *E. coli* colonization and potentially prevent recurrent symptomatic episodes [29]. In postmenopausal women, low-dose estriol may additionally contribute to prophylaxis by restoring an unfavorable vaginal pH for uropathogens [30]. Thus, evidence concerning vaginal dysbiosis and microbiota modulation should be interpreted separately from direct evidence of clinical prevention of recurrent uncomplicated UTI.

### 3.4. The Physiology of Immune Response in UTIs

The host immune response is an important determinant of susceptibility to recurrent UTIs [31]. Women are more prone to UTIs than men, but men more often suffer from complicated UTIs. In a mouse model, only male and testosterone-treated mice developed chronic infection, whereas female mice showed higher IL-17 expression and increased numbers of γδ T cells and group 3 innate lymphoid cells in the bladder [32]. Mucosal-associated invariant T (MAIT) cell numbers are similar in patients with recurrent UTIs and healthy controls, but MAIT cells in patients with recurrent UTIs exhibit altered activation, including increased T-bet expression and a higher activation threshold [33]. Overall, these findings provide mechanistic insight into host susceptibility and immune responses, but evidence from animal models or immunological studies should be distinguished from direct clinical evidence supporting a preventive intervention in women with recurrent uncomplicated UTIs.

**Figure 1:**
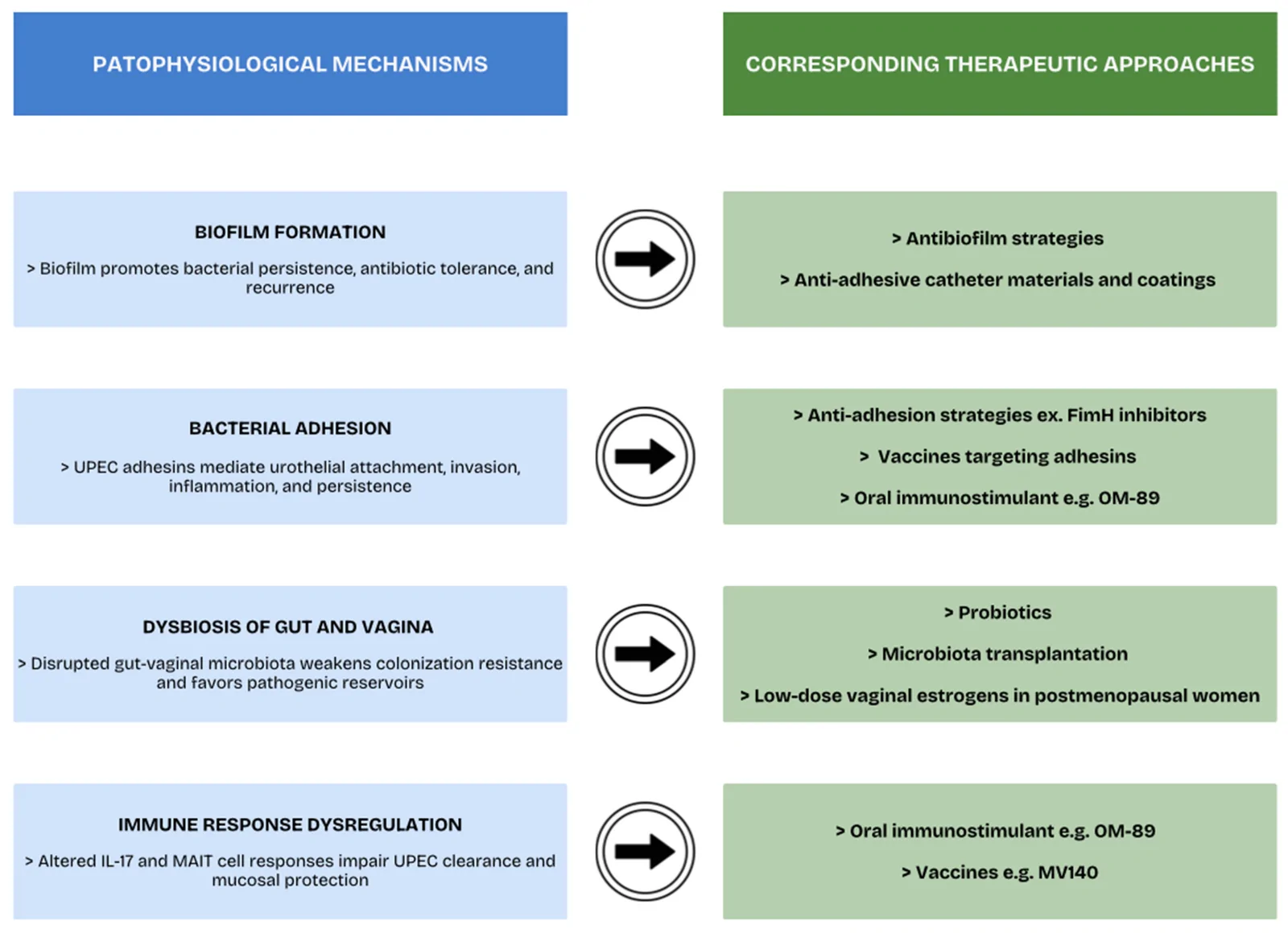
Pathophysiological background of recurrent UTIs with corresponding therapeutic approaches. Abbreviations: UPEC—Uropathogenic *Escherichia coli*; IL—interleukin; MAIT—Mucosal-associated invariant T.

Pathophysiological background of recurrent UTIs with corresponding therapeutic approaches summarized in Figure 1.

## 4. Limitations of Conventional Antibiotic Therapy

There are distinct strategies for managing recurrent UTIs: one focuses on administering antibiotics episodically when symptoms and infections occur, while the other relies on continuous suppressive therapy to inhibit bacterial growth. In a randomized trial directly addressing which strategy is better, continuous prophylaxis achieved superior symptom suppression compared to patient-administered therapy (0.2 vs. 2.2 recurrences per patient-year) [34]. However, this continuous approach carries potential adverse effects, as long-term antibiotic use can lead to significant clinical consequences. Long-term antibiotic exposure may promote the selection of antimicrobial-resistant organisms, alter the gut and vaginal microbiota, and increase the risk of antibiotic-associated adverse events. In a randomized study, prolonged trimethoprim-sulfamethoxazole prophylaxis was associated with a substantial rise in antimicrobial resistance among *Escherichia coli* isolates, followed by a reduction in resistance after treatment cessation [35]. Similarly, the ALTAR randomized trial reported a higher proportion of antimicrobial-resistant *E. coli* isolates among women receiving daily antibiotic prophylaxis compared with those receiving methenamine hippurate [36]. That being said, self-start therapy remains effective, safe, and economical, particularly given that women were able to accurately diagnose a UTI based on symptoms in 92% of cases [37]. A prominent retrospective study in this field, conducted by Carl Llor et al., compared patients receiving continuous suppressive therapy with those receiving episodic treatment (in which antibiotics were administered only at symptom onset). The study tracked severe outcomes, including COVID-19, pyelonephritis, pneumonia, septicemia, influenza, and death. These adverse events were observed in 12.6% of women treated with suppressive prophylactic antibiotic therapy, compared to only 6.4% in the episodic treatment group. However, because this was a retrospective observational comparison, these findings may be influenced by confounding by indication and should not be interpreted as evidence of a causal relationship. The most significant correlation was observed with pyelonephritis, where the risk increased by 64 to 133 percent. The second most common severe adverse effect was septicemia, exhibiting a risk increase of 13 to 59 percent. The most frequently prescribed drug to manage UTIs was fosfomycin [38]. Overall, these findings represent associations rather than demonstrated causal effects of suppressive antibiotic therapy. A higher incidence of complications was observed in patients undergoing suppressive therapy, although differences in baseline risk between the groups may have contributed to this finding. Another effective therapeutic approach for healthy young women prone to recurrent urinary tract infections is postcoital prophylaxis. This method has shown promising results, substantially reducing the incidence of UTIs from 3.6 to 0.3 episodes per patient-year [39].

Several theories attempt to explain the higher incidence of adverse effects associated with continuous antibiotic therapy. One prominent hypothesis focuses on the vaginal microbiota and the role of local microorganisms [40,41,42,43]. The *Lactobacillus* population can be adversely affected by various factors, including estrogen depletion during menopause, recurrent UTIs, and notably, the use of antimicrobial agents [44]. Consequently, antibiotic therapy can deplete *Lactobacillus* colonies, inadvertently increasing the risk of subsequent UTIs. This depletion impairs several protective mechanisms, such as the production of hydrogen peroxide [45], competitive inhibition of uropathogen adhesion [46], maintenance of an acidic environment via lactic acid production, and the secretion of type 1 interferons, which decreases the intracellular bacterial burden [40]. Notably, *Lactobacillus crispatus* exhibits the most robust protective effect.

Another critical aspect is the proliferation of antibiotic-resistant strains within the patient’s microflora. According to the updated 2025 AUA/CUA/SUFU guidelines, fluoroquinolones and cephalosporins are highlighted as frequently prescribed antibiotics that significantly contribute to multidrug resistance. Specifically, their widespread use has been linked to the emergence of resistant pathogens, including methicillin-resistant *Staphylococcus aureus* (MRSA), ESBL-producing *Klebsiella pneumoniae*, and β-lactam-resistant *Acinetobacter* species. Researchers also highlight poor patient adherence; individuals frequently adjust dosages independently or prematurely discontinue their treatment courses [47]. That being said, not all antimicrobial agents inevitably lead to increased resistance [48].

## 5. Immunoprophylaxis and Vaccines

The growing problem of antibiotic resistance has led to a shift in approaches to preventing recurrent urinary tract infections in women, with greater emphasis on harnessing the patient’s immune system. These strategies intend to activate both the innate and adaptive immune responses. The most extensively studied groups of preparations include oral immunostimulants and next-generation mucosal vaccines [49,50,51].

### 5.1. Oral Immunostimulants

Of the available oral immunostimulatory preparations, OM-89 is among the best documented for clinical efficacy. It is a bacterial lysate derived from 18 of the most frequently occurring UPEC [52]. After oral administration, the preparation activates the lymphoid tissue associated with the gastrointestinal mucosa (GALT). This occurs through stimulation of Toll-like receptors (TLRs) on macrophages, which triggers the Nuclear factor of kappa light-chain enhancer of B cells (NF-κB) signaling cascade and induces the production of pro-inflammatory cytokines. B lymphocytes activated in GALT differentiate into plasma cells, which then migrate via the bloodstream to mucosal surfaces throughout the body, including the urinary bladder. There, they secrete secretory immunoglobulin A (sIgA), which may inhibit the adhesion and invasion of uropathogens, thereby increasing the urinary tract’s resistance [53,54,55].

Available clinical data suggest that OM-89 may reduce recurrent UTI frequency in women, although the certainty of the evidence is limited by heterogeneity and methodological limitations across the available trials. In the systematic review and meta-analysis by Beerepoot et al., comprising a total of 891 participants, four randomized placebo-controlled trials showed a pooled risk ratio (RR) of 0.61 (95% CI: 0.48–0.78; *p* < 0.05) for UTI recurrence, with a safety profile compared to placebo [56]. The included trials evaluated adults with recurrent UTIs and generally assessed recurrence over approximately 6 months, although study populations, outcome definitions, and follow-up periods varied across individual trials. Thus, the pooled estimate supports a potential preventive effect but should be interpreted in the context of the relatively heterogeneous and older evidence base. A prospective cohort study by Kim et al., involving 34 patients with chronically recurrent cystitis, reported a marked reduction in recurrences: the immunostimulant was administered once daily for three months, followed by six months of follow-up, and the mean number of recurrences fell from 4.26 during the six months before treatment to 0.35 after the course (*p* < 0.001), with 82.4% of women becoming infection-free. However, this uncontrolled study provides lower-certainty evidence than the randomized data [57]. Taken together, OM-89 has supportive randomized evidence for reducing rUTI recurrence, but the certainty of evidence should not be considered equivalent to that of a contemporary, large, multicenter clinical trial.

### 5.2. Next-Generation Vaccines

Alongside the development of bacterial lysates, the limitations of the oral route were recognized, as was the need to broaden the antigenic spectrum, finally leading to the development of next-generation polybacterial vaccines. An example of such a preparation is MV140 (Uromune^®^ ((Inmunotek S.L., Alcalá de Henares, Spain)) contains whole, inactivated cells of four bacteria: *E. coli*, *K. pneumoniae*, *P. vulgaris*, and *E. faecalis*, all of which are common uropathogens implicated in recurrent UTIs [58].

The vaccine is administered sublingually at a dose of two sprays per day. It is absorbed through the sublingual mucosa, where a dense network of dendritic cells (DCs) plays an essential role in linking innate and adaptive immune responses and enabling the induction of mucosal immunity [58]. Earlier mucosal vaccine strategies included vaginal preparations in the form of suppositories; however, due to limited patient acceptance and local adverse effects, such as mucosal irritation, the sublingual route has become more widely adopted [56,59].

A multicenter, double-blind, randomized, placebo-controlled trial in Spain and the United Kingdom included 240 women aged 18–75 years with rUTIs, randomized to MV140 for 3 or 6 months or placebo for 6 months and followed for 12 months. The median number of UTI episodes was 3.0 (IQR 0.5–6.0) with placebo versus 0.0 (IQR 0.0–1.0) with MV140 (*p* < 0.001), while UTI-free rates were 25% (95% CI 15–35%) versus 56% (95% CI 44–67%) and 58% (95% CI 44–67%) after 3 and 6 months of MV140, respectively [60]. Although these results provide randomized evidence of benefit, the study was of modest size and duration, and the findings should therefore be interpreted as supportive rather than definitive evidence of long-term preventive efficacy. Subsequent secondary analyses demonstrated that in the group receiving MV140, there was a significant reduction in UTIs symptom severity (102.2 vs. 194.2; *p* = 0.0002), an almost threefold shortening of antibiotic treatment duration (12.4 vs. 28.7 days; *p* = 0.0001), and a significant improvement in quality of life across all SF-36 domains (overall difference: 15.7; 95% CI: 8.80–22.64; *p* < 0.0001) [61].

Another key piece of evidence is the prospective, multicenter cohort study by Ramírez Sevilla et al. (2024), which involved 1104 women with uncomplicated rUTIs and at least three UTI episodes at baseline (mean age, 72 years). The study was uncontrolled and assessed outcomes at 3, 6, and 12 months after immunoprophylaxis. Efficacy was predefined as having 0–2 UTI episodes during follow-up. After a three-month course, the predefined efficacy criterion (0–2 UTI episodes throughout follow-up) was met by 95.8% of patients; at 12 months, this proportion was 57.6%, and 64.7% among women with a milder disease history (3–4 UTI episodes per year). Because there was no placebo or untreated comparator, these results cannot establish the causal preventive efficacy of MV140, and the findings should be considered lower-certainty evidence than those from the randomized trial [62].

Overall, OM-89 and MV140 have supportive clinical evidence for reducing rUTI recurrence, including randomized evidence for both interventions, but the certainty of evidence varies and should be interpreted in light of study size, follow-up duration, heterogeneity, and methodological limitations. Their regulatory availability also varies between countries, and evidence of clinical efficacy should not be equated with universal regulatory approval or routine availability. Clinical use may also depend on product availability and appropriate patient selection.

## 6. Microbiome-Based Therapies

The vaginal microbiome of a healthy woman is predominantly composed of *Lactobacillus* spp., which maintain a low pH and limit pathogen colonization by producing lactic acid and other metabolites. These characteristics support homeostasis and protect against bacterial and fungal infections [63]. Dysbiosis of the urogenital tract occurs when the proportion of *Lactobacillus* decreases, and anaerobic bacteria increase, leading primarily to bacterial vaginosis (BV) and its potential complications [64,65].

Following the success of fecal microbiota transplantation in treating *C. difficile* infection, researchers have begun investigating the vaginal microbiome. Clinical trials are currently underway to evaluate approaches to support the restoration and modulation of a healthy vaginal microbiome in states of dysbiosis [64]. Although numerous clinical studies and meta-analyses indicate potential benefits of probiotic therapy in treating infections and microbiome disturbances, its clinical efficacy and impact on reducing recurrence remain uncertain. This is largely due to strain heterogeneity, differences in routes of administration, inconsistent study quality, and challenges in defining a standardized clinical dose [66,67]. In dysbiosis-targeted therapy, probiotics may act through lactic acid production, competition for adhesion sites, and immune-modulation mechanisms relevant to both the prevention and treatment of BV and fungal infections [63].

A study involving 36 asymptomatic women with vaginal dysbiosis evaluated oral probiotic therapy (*L. acidophilus*, *L. rhamnosus*, and *L. reuteri*) administered for six weeks. Participants were classified according to the Nugent score (NS) into Low-NS (n = 26) and High-NS (n = 10) groups. The results showed that 60% of women with a high Nugent score shifted toward a lower Nugent score and showed increased vaginal colonization, while four remained in the High-NS group. Among women with low NS, 11.5% shifted to a high-NS profile characterized by increased anaerobic bacteria [64]. Importantly, this study did not assess symptomatic UTI or rUTI recurrence; therefore, its findings provide evidence of vaginal microbiota modulation instead of rUTI prevention.

Meta-analyses and systematic reviews consistently report improvements in clinical and microbiological parameters following probiotic therapy with *Lactobacillus* strains [65,68]. However, despite promising findings, many commercial probiotics fail to achieve stable vaginal colonization or demonstrate clear clinical efficacy. The limited number of studies and their inconclusive results highlight the need for larger, well-designed clinical trials [65].

Vaginal microbiota transplantation (VMT) is a promising therapeutic strategy to restore *Lactobacillus* dominance in patients with recurrent dysbiosis. In the scoping review by Luo H [69], 12 studies were included: seven published (three human studies and four animal studies) and five ongoing clinical trials. Across 36 women, VMT restored a *Lactobacillus*-dominated microbiome and improved symptoms of BV and other forms of urogenital dysbiosis. In animal models, reductions in inflammatory markers were observed, and two mouse studies demonstrated increased *Lactobacillus* abundance following VMT [65]. However, these studies did not evaluate prevention of recurrent UTI as a clinical endpoint. Therefore, evidence supporting VMT for rUTI prevention is currently indirect and preliminary, and dedicated clinical trials in women with rUTI are required before conclusions regarding preventive efficacy can be drawn.

In summary, evidence supporting *Lactobacillus*-based probiotics for preventing rUTI recurrence remains heterogeneous and inconclusive. Although some randomized clinical studies have demonstrated a reduction in symptomatic rUTI recurrence, the available evidence does not establish consistent efficacy across probiotic strains and routes of administration. Vaginal microbiota transplantation represents a promising but still experimental approach; current evidence mainly comes from studies of vaginal dysbiosis, bacterial vaginosis, and does not directly assess rUTI prevention. Dedicated, well-designed clinical trials in women with rUTIs are needed before preventive efficacy can be established.

## 7. Bacteriophage Therapy

Bacteriophage (phage) therapy is an emerging approach that has gained renewed interest, particularly in the context of multidrug-resistant urinary tract infections. Several phage-based therapies are currently being evaluated in clinical studies, including LBP-EC01. Bacteriophages are highly specific viruses that infect and lyse susceptible bacteria, offering the potential for targeted pathogen elimination while preserving the host microbiota. This characteristic is particularly relevant in rUTIs, where repeated antibiotic exposure may contribute to dysbiosis [69].

New clinical data, observed on 100 patients, suggest that phage therapy may be associated with microbiological and clinical improvement in approximately 77.2% of cases, with complete bacterial eradication reported in 61.3% of patients [69,70]. Follow-up studies have reported low rates of reinfection after treatment. However, further studies are needed to confirm these findings.

These findings provide preliminary clinical evidence regarding the use of personalized bacteriophage therapy in bacterial infections, but they do not establish its efficacy in preventing recurrent uncomplicated UTIs. Clinical or microbiological improvement and bacterial eradication should not be interpreted as equivalent to a reduction in subsequent UTI recurrence. The preventive role of bacteriophage therapy therefore remains investigational.

Routes of administration include intravesical instillation, commonly used for UTIs, as well as oral administration. In vivo on mice studies have also investigated phage-antibiotic synergy (PAS). For example, in a study using the *Acinetobacter baumannii* AB900 strain, combination therapy with phage øFG02 and ceftazidime resulted in a greater reduction in bacterial burden than phage monotherapy [71].

Phage immunogenicity may also limit therapeutic efficacy, as neutralizing antibodies can develop relatively rapidly following administration [72,73]. However, researchers emphasize that this immune response is highly dependent on patient susceptibility and the specific route of administration. Notably, clinical observations by Łusiak-Szelachowska et al. suggest that oral intake may offer the optimal efficacy profile by minimizing the rapid production of neutralizing antibodies compared with other systemic routes [73].

Overall, bacteriophage therapy shows promising potential as a targeted approach to rUTIs, particularly in the context of antimicrobial resistance. However, the available evidence remains limited and heterogeneous, with important concerns regarding phage resistance, immunogenicity, treatment standardization, and patient selection. Further well-designed clinical trials are required before phage therapy can be considered for routine management of rUTIs.

## 8. Other Emerging Approaches

In addition to established strategies such as immunoprophylaxis and antibiotic therapy, several other approaches are being investigated for the prevention and treatment of rUTIs, including antimicrobial peptides (AMPs), nanotechnology, molecularly targeted therapies, and precision medicine strategies [74,75].

AMPs are cationic molecules that are part of innate immunity, providing protection against uropathogens on mucosal surfaces. In the urinary tract, the primary AMPs include alpha- and beta-defensins, cathelicidins (particularly LL-37), ribonucleases, and Tamm–Horsfall protein (uromodulin) [76]. These peptides interact with bacterial membranes and may cause membrane disruption and pathogen death [77]. In addition, the cathelicidin LL-37 has immunomodulatory properties and may promote the recruitment of immune cells to sites of inflammation [78]. Human evidence is mainly observational or translational, showing altered AMP expression in women with UTI or rUTI, whereas direct AMP-based treatments have so far been evaluated predominantly in experimental models [79,80]. No randomized clinical trials have demonstrated that direct AMP administration prevents rUTI in women.

Estrogens may also enhance urothelial immune mechanisms by increasing AMP expression, potentially limiting the formation of intracellular bacterial communities (IBCs) [81]. Urinary AMP levels have therefore been investigated as potential biomarkers of rUTI recurrence. Cathelicidin expression increases following exposure to *E. coli*, whereas persistent LL-37 deficiency after a previous infection may be associated with an increased risk of recurrence [78]. In contrast to direct AMP-based therapies, vaginal estrogen is supported by randomized controlled trials and meta-analytic evidence for the prevention of rUTI in postmenopausal women. A meta-analysis of randomized trials demonstrated a significant reduction in recurrence with vaginal estrogen compared with placebo (RR 0.42, 95% CI 0.30–0.59; [82], with additional support from more recent randomized trials [30].

Nanotechnology may facilitate intravesical drug delivery by addressing limitations of conventional therapies, including rapid urinary clearance and the glycosaminoglycan (GAG) barrier [83]. In preclinical studies, organic nanoparticles loaded with ciprofloxacin showed greater activity against UPEC and more effective inhibition of biofilm formation than the free antibiotic [84]. Similarly, nanogels with cell-penetrating peptides (CPPs) may enable direct delivery of gentamicin to the urothelium, with preclinical studies suggesting a substantial reduction in IBCs [83]. Among inorganic carriers, selenium and zinc oxide nanoparticles have also been investigated for their ability to induce oxidative stress in pathogens [85]. Another experimental approach involves biodegradable platelet-like nanostructures designed to improve interaction with colonizing bacteria and the urothelium [86]. Despite these encouraging findings, the evidence for nanoparticle-based treatment of UTIs remains predominantly preclinical, with the highest level of direct therapeutic evidence derived from in vitro experiments and animal models [83,84,85,86]. In particular, there is currently insufficient evidence that nanoparticle-based interventions reduce symptomatic recurrence in women with rUTIs.

Host-directed therapies (HDTs) represent an alternative approach that strengthens the urothelium’s endogenous resistance rather than directly targeting pathogens. Potential targets include the urothelial barrier, innate immune pathways such as TLRs, and epigenetic regulation of immune gene expression [74,76]. Although most HDTs for rUTI remain at the preclinical stage, some have now progressed to clinical evaluation. Experimental studies have shown that targeting dysregulated host inflammatory responses, particularly COX-2- and IL-1-mediated pathways, can reduce bladder inflammation and bacterial burden and, in animal models, decrease susceptibility to recurrent cystitis [87,88]. More recently, a randomized, open-label Phase 2 trial evaluated the IL-1 receptor antagonist anakinra in 30 women with recurrent cystitis. Anakinra improved cystitis symptoms and was non-inferior to nitrofurantoin, while recurrence rates during the 6-month follow-up were lower than during the 6 months before enrolment [89]. However, the study was small and conducted at a single centre, and these findings require confirmation in larger randomized trials. Anakinra is not currently approved for the treatment or prevention of rUTI; therefore, its use for this indication would be off-label. At present, the available evidence is insufficient to support HDTs as an established or routinely prescribed treatment for rUTI.

Biofilm formation represents another important therapeutic target, as biofilm-associated UPEC may tolerate substantially higher antibiotic concentrations than planktonic bacteria and contribute to recurrent infection [90]. Anti-adhesive molecules, including mannose-based compounds, may interfere with UPEC attachment to host tissues, while synthetic inhibitors targeting fimbrial adhesins such as FimH have been investigated as potential anti-biofilm strategies [91]. Mannose-based anti-adhesive strategies have focused primarily on synthetic mannosides and related compounds designed to inhibit FimH, the type 1 fimbrial adhesin that mediates UPEC attachment to the urothelium. These compounds have shown potent inhibition of UPEC adhesion in vitro and significant reductions in urinary and bladder bacterial burden in murine UTI models, including after oral administration [92,93]. FimH-targeting mannosides have also been shown to reduce intestinal UPEC reservoirs in experimental models, suggesting a potential role in preventing recurrent infection [94]. However, the evidence remains predominantly preclinical. A small pilot study in six healthy volunteers demonstrated urinary excretion of the FimH inhibitor 1-deoxymannose following oral administration but did not assess its efficacy in the treatment or prevention of rUTI [95]. Thus, although synthetic mannose-based FimH inhibitors represent a promising anti-adhesive strategy, clinical evidence is currently insufficient to support their routine use.

rUTIs are increasingly recognized as a heterogeneous condition in which susceptibility may be influenced by immune responses, the microbiome, genetic factors, and hormonal status. Urinary biomarkers, including sIgA, AMPs, and cytokine profiles, have been investigated to identify women at increased risk of recurrence. The potential genetic contribution is illustrated by studies of the DEFA1A3 polymorphism, in which copy-number variation has been associated with UTI risk. Such findings raise the possibility of identifying patients with reduced AMP expression who could benefit from targeted supplementation or immunostimulation.

Overall, emerging approaches including AMPs, nanotechnology, host-directed therapies, and anti-biofilm strategies show promising potential, particularly based on mechanistic and preclinical findings. However, most remain at an early experimental stage, with evidence derived predominantly from in vitro studies, animal models, or small clinical studies. Further well-designed clinical trials are needed to establish their efficacy, safety, and appropriate patient selection before routine clinical implementation.

## 9. Future Directions and Clinical Implications

Future strategies for preventing recurrent urinary tract infections increasingly focus on non-antibiotic approaches that target multiple pathogenic mechanisms of uropathogens, with the aim of reducing antibiotic resistance. However, the long-term efficacy of many of these interventions remains uncertain, highlighting the need for further well-designed clinical trials to confirm their effectiveness and durability [96,97].

Vaccinations and plant-based therapies are among the most promising strategies. Although vaccines may reduce the burden of rUTIs, their effectiveness may be limited by host-related factors, pathogen replacement within the urogenital niche, and the complexity and cost of vaccine development [96]. Similarly, medicinal plants represent a potential source of novel antibacterial agents, but their mechanisms of action and clinical efficacy require further investigation before they can be implemented in routine practice [96].

Furthermore, a topic worth exploring regarding future therapeutic approaches is the use of D-mannose as a potential non-antibiotic intervention for the prevention of recurrent urinary tract infections in women. Although previous studies have suggested a beneficial effect of D-mannose in reducing rUTI recurrence [98], these findings were not confirmed in a recent large randomized, placebo-controlled trial, which found no significant reduction in subsequent clinically suspected UTIs among women receiving daily D-mannose [99]. These findings suggest that D-mannose alone should not currently be considered an effective prophylactic strategy for rUTIs [99]. Nevertheless, further research may help determine whether D-mannose could provide benefits in specific patient populations or when combined with other non-antibiotic preventive strategies.

**Figure 2:**
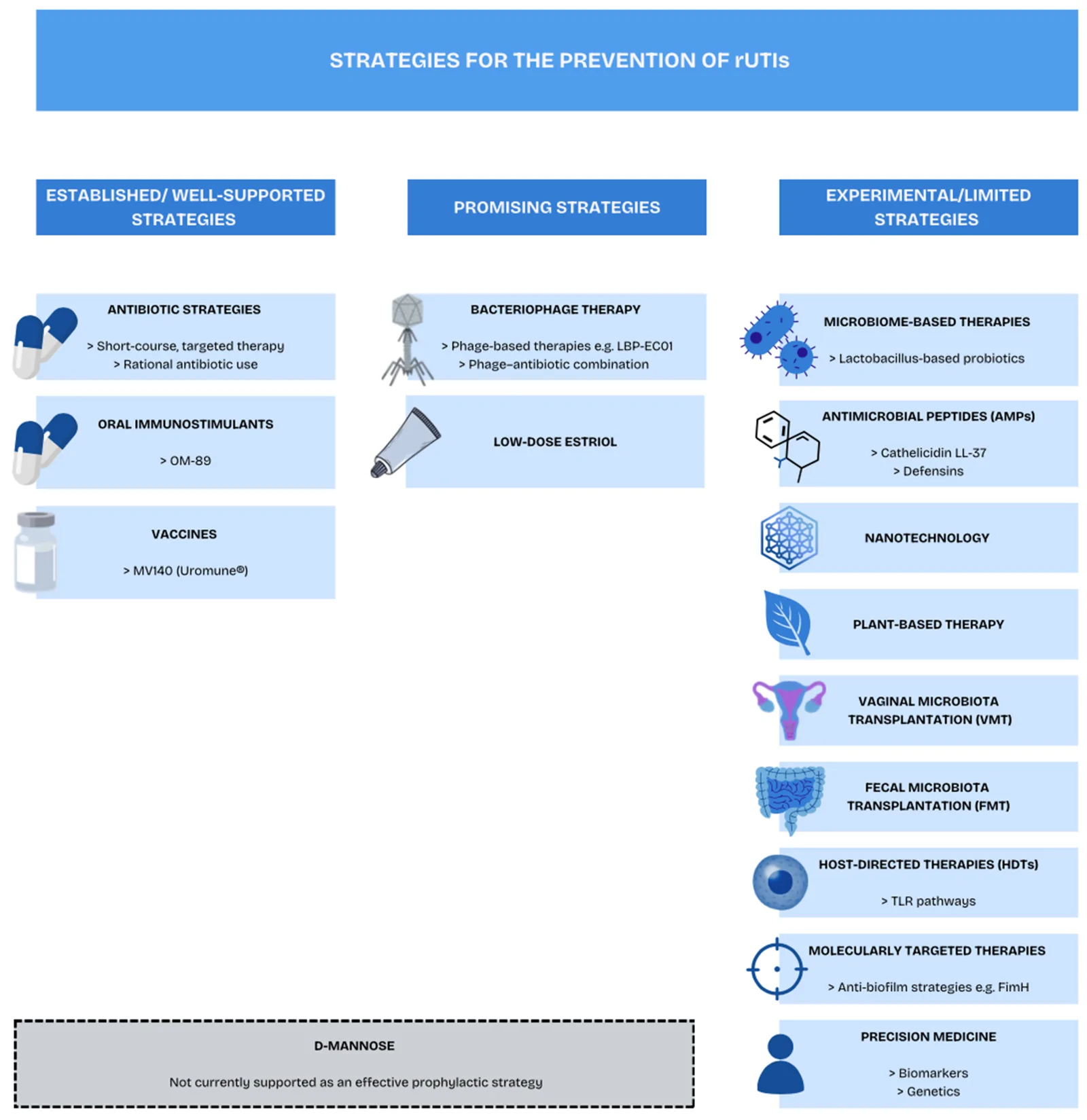
Strategies for the prevention of rUTIs divided into three categories: established/well-supported, promising, and experimental. Abbreviations: rUTIs—Recurrent urinary tract infections; AMPs—Antimicrobial peptides; VMT—Vaginal microbiota transplantation; FMT—Fecal microbiota transplantation; HDTs—Host-directed therapies; TLR—Toll-like receptor. **Note:** Evidence categories reflect the overall level and maturity of evidence described in this narrative review and do not represent a formal risk-of-bias or GRADE assessment.

The main preventive strategies, together with their study designs, sample sizes, main findings and limitations are summarized in Table 2. The classification of these strategies according to the level of available evidence is presented in Figure 2.

## 10. Conclusions

Contemporary management of recurrent UTIs continues to rely on antibiotic therapy, while selected non-antibiotic strategies have gained increasing attention as preventive approaches. Clinically supported interventions include immunoprophylaxis and selected vaccines, whereas microbiome-targeted therapies and bacteriophage therapies remain promising but exploratory and require further validation before their preventive efficacy can be established. The strength of clinical evidence varies between interventions and study designs. Other approaches, including AMPs, host-directed therapies, nanotechnology-based drug delivery systems, and anti-biofilm strategies, remain largely preclinical or investigational. Vaginal microbiota transplantation, FimH-targeted approaches, and anakinra also remain exploratory and should not be considered established preventive interventions in women with recurrent uncomplicated UTIs. Evidence from studies of vaginal dysbiosis or bacterial eradication alone should not be interpreted as direct evidence of preventive efficacy against recurrent UTIs. Advances in understanding host immune responses and the role of the intestinal and vaginal microbiota may further support the development of personalized prevention. Although several emerging interventions, including plant-derived compounds, show promise, others, such as D-mannose, have not demonstrated consistent clinical benefit and require further high-quality clinical studies to clarify their role in preventing rUTIs before consideration for routine clinical implementation. Overall, the available evidence for several emerging strategies remains indirect, experimental, or preclinical, and their potential role in rUTI prevention requires confirmation in well-designed clinical studies.

## Figures and Tables

**Table 1:** Databases and search terms used, and number of identified records.

Key Terms/Database	PubMed	Web of Science	Scopus	Total
**“recurrent urinary tract infections in women” AND “antibiotic prophylaxis”**	221	365	427	1013
**“recurrent urinary tract infections in women” AND “immunoprophylaxis”**	15	119	22	156
**“recurrent urinary tract infections in women” AND “urinary microbiome”**	83	189	94	366
**All identified articles**				1535
**Records excluded during the selection process (duplicates and irrelevant records)**				1435
**Articles included in the review**				100

**Table 2:** Characteristics and level of evidence of studies evaluating strategies for the prevention of recurrent urinary tract infections. Abbreviations: UTI—urinary tract infection; RR—risk ratio; CI—confidence interval; RCT—randomized controlled trial; rUTIs—recurrent urinary tract infections; UPEC—uropathogenic *Escherichia coli*.

Strategy	Study Design/Evidence Type	Population	Comparator	Sample Size	Follow-Up	Recurrence Definition/Outcome	Effect Estimate (95% CI)	Directness of Evidence	Major Limitations
**Antibiotic prophylaxis—continuous**	Randomized crossover trial; additional observational cohort evidence for harms	Women with recurrent UTI	Patient-administered single-dose therapy; observationally, no suppressive therapy	38 (Wong); 36,170 (Llor cohort)	6 months per treatment period in Wong; up to 100 months in Llor	Wong: symptomatic UTI episodes/patient-year. Llor: severe outcomes, not rUTI recurrence	0.2 vs. 2.2 episodes/patient-year, *p* < 0.001; no CI reported. Llor: severe-outcome HR 1.50 (95% CI 1.33–1.68)	Direct for recurrence prevention; indirect/observational for severe harms	Small, old crossover trial; Llor study is retrospective and susceptible to confounding by indication; severe-outcome association should not be interpreted causally.
**Self-start antibiotic therapy**	Clinical prospective study	Women with recurrent UTI	No concurrent control	34 women	Variable; 28 women contributed 355 patient-months; individual follow-up up to 103 months	Symptomatic episodes and culture-confirmed UTIs during self-treatment	2.8 symptomatic episodes/patient-year; 2.3 infections/patient-year; 92% of symptomatic episodes responded clinically; no CI reported	Direct for patient-initiated treatment, but not a controlled prophylaxis comparison	Uncontrolled study; treatment strategy rather than prophylactic intervention; relies on patient recognition of symptoms.
**Postcoital antibiotic prophylaxis**	Randomized, double-blind, placebo-controlled trial	Healthy young women prone to recurrent UTI	Postcoital placebo	27 randomized (16 active, 11 placebo)	>6 months	Recurrent UTI episodes; infection rate per patient-year	0.3 vs. 3.6 episodes/patient-year; no CI reported	Direct	Very small, older study; restricted to healthy young women with recurrent UTIs associated with sexual activity.
**OM-89**	Systematic review and meta-analysis of RCTs; prospective uncontrolled cohort	Adults/women with recurrent UTI; cohort specifically women with chronic recurrent cystitis	Placebo in pooled RCTs; no comparator in cohort	891 in 4 RCTs; cohort n = 34	RCTs: approximately 20 weeks–12 months; cohort: 3-month treatment + 6-month follow-up	Proportion with ≥1 recurrent UTI/number of recurrences	Meta-analysis: RR 0.61 (95% CI 0.48–0.78). Cohort: 4.26 → 0.35 recurrences, *p* < 0.001	Direct for OM-89 and rUTI recurrence	Older heterogeneous trials; moderate methodological quality; cohort uncontrolled and small
**MV140 (Uromune^®^)**	Multicenter randomized, double-blind, placebo-controlled trial; prospective multicenter cohort	Women with recurrent uncomplicated UTI; cohort: 1104 women, ≥3 UTIs at baseline	Placebo in RCT; no untreated comparator in cohort	240 in RCT; 1104 cohort	RCT: 12-month study, primary efficacy period 9 months; cohort: 3, 6 and 12 months	RCT: number of UTIs and UTI-free status; cohort: efficacy defined as 0–2 UTIs during follow-up	RCT: median 0 vs. 3 UTIs; UTI-free 56% (95% CI 44–67%)/58% (44–67%) vs. 25% (15–35%) placebo. Cohort: efficacy 95.8% at 3 mo; 57.6% at 12 mo	Direct for RCT; direct but lower-certainty for cohort	RCT modest sample; long-term efficacy uncertain; cohort uncontrolled and therefore cannot establish causality.
***Lactobacillus*** **-based probiotics**	Randomized, double-blind, placebo-controlled trial; systematic reviews/meta-analyses	Premenopausal women with recurrent UTI	Placebo; oral and/or vaginal placebo	174 women in direct rUTI RCT	4-month intervention; 12-month follow-up	Symptomatic UTI recurrence; number of symptomatic UTIs and time to first UTI	At 4 months: mean recurrences 2.10 placebo vs. 1.06 vaginal probiotic vs. 1.07 combined probiotic; no CI reported	Direct for the 2024 RCT; broader probiotic evidence heterogeneous	Strain and route heterogeneity; different preparations/doses; previous literature includes studies targeting vaginal dysbiosis rather than rUTI.
**Vaginal microbiota transplantation (VMT)**	Scoping review including human and animal studies; ongoing clinical trials	Women with vaginal dysbiosis/BV; animal models; not specifically women with rUTI	No standardized comparator	36 women across published human studies; 3 human + 4 animal published studies; 5 ongoing trials	Variable; no standardized follow-up	Primarily vaginal dysbiosis/BV outcomes; rUTI recurrence was not assessed	No valid rUTI effect estimate or CI	Indirect for rUTI prevention	Small human samples; heterogeneous procedures; animal evidence; no standardized donor/preparation protocols; absence of direct rUTI endpoint.
**Bacteriophage therapy**	Retrospective multicenter/multinational observational case series; additional experimental evidence	Patients with difficult-to-treat infections at multiple sites; not rUTI-specific	No standardized comparator; concomitant antibiotics used in many cases	100 patients; 114 targeted infections	Variable, real-world follow-up; not standardized for rUTI recurrence	Clinical improvement and bacterial eradication; no standardized rUTI recurrence endpoint	Clinical improvement 77.2% (88/114); eradication 61.3% (65/106). No rUTI-specific effect estimate. OR for eradication without concomitant antibiotics 0.30 (95% CI 0.127–0.749)	Very indirect for prevention of recurrent uncomplicated UTI in women	Limited Mixed infection sites and pathogens; uncontrolled; heterogeneous phage preparations; resistance and immunogenicity; findings cannot be interpreted as evidence of rUTI prevention.
**Low-dose estriol**	Randomized, double-blind, placebo-controlled multicenter trial; supported by meta-analysis of RCTs	Postmenopausal women with genitourinary syndrome of menopause	Identical moisturizing vaginal gel/placebo	108 randomized	24 weeks	Number of UTI episodes; recurrence/time to first recurrence	IRR 0.739 (95% CI 0.6459–0.8455); UTI incidence 32.34 vs. 43.76/100 women-year	Direct	Short follow-up; specific postmenopausal/GSM population; trial sample modest. Meta-analysis also showed RR 0.42 (95% CI 0.30–0.59) for vaginal estrogen.
**Antimicrobial peptides (AMPs)**	Mechanistic, in vitro, animal/preclinical studies; observational/translational human evidence	UPEC/experimental models; women with UTI/rUTI in observational biomarker studies	Usually none	Not applicable as a single pooled population	Not applicable/variable	AMP expression, bacterial burden or inflammatory responses; no validated clinical recurrence endpoint for AMP administration	No clinical preventive effect estimate/CI	Indirect/preclinical	No randomized clinical trial demonstrating that direct AMP administration prevents rUTI in women; predominantly experimental evidence.
**Nanotechnology**	In vitro and animal/preclinical experimental studies	UPEC and experimental UTI models	Free antibiotic or untreated/control experimental groups depending on study	Not applicable as a single population	Experimental/variable	Bacterial burden, biofilm or intracellular bacterial communities; no clinical rUTI endpoint	No clinical effect estimate/CI	Preclinical/indirect	No clinical evidence demonstrating reduction in symptomatic rUTI recurrence; heterogeneous nanoparticle systems and experimental models.
**Plant-based therapies**	Clinical trials/meta-analyses and preclinical/mechanistic evidence	Heterogeneous populations depending on botanical intervention	Variable; placebo/standard treatment in individual trials	Not applicable as a single population	Variable	UTI incidence/recurrence depending on intervention; no single standardized recurrence definition	No single pooled estimate applicable to the strategy as a whole	Variable; direct for selected botanicals, otherwise indirect	Heterogeneous products, doses and preparations; evidence varies markedly between cranberry, herbal medicines and other compounds; current review itself does not provide one strategy-level effect estimate.
**Fecal microbiota transplantation (FMT)**	Retrospective observational cohort; mechanistic/preclinical evidence	Patients undergoing FMT for recurrent *C. difficile* infection who also had rUTI	Historical pre-FMT period; no concurrent control	11 patients with rUTI	2 years before vs. 2 years after FMT	rUTI defined as ≥2 UTIs/6 months or ≥3/12 months	3.7 → 0.27 UTIs over comparable 2-year periods, *p* = 0.001; median time to recurrence 19.6 months (95% CI 15.2–23.9)	Direct but very low-certainty clinical evidence	Very small uncontrolled cohort; FMT was performed for *C. difficile*, not specifically rUTI; retrospective design and no comparator.
**Host-directed therapies (HDTs)**	Mechanistic/preclinical studies plus randomized open-label phase 2 trial of anakinra	Women with recurrent acute cystitis and current acute episode; animal models for other HDTs	Nitrofurantoin in anakinra trial; none in preclinical studies	30 women in phase 2 trial (20 anakinra, 10 nitrofurantoin)	5-day treatment; follow-up days 15, 30 and 6 months	Recurrences during 6-month follow-up; comparison with 6-month pre-enrolment history	Recurrences decreased in both arms vs. pre-enrolment period; no rUTI effect estimate with CI reported	Direct but preliminary for anakinra; otherwise preclinical/indirect	Single-centre, small sample, open-label; comparison partly relies on pre-enrolment history; anakinra is not established/approved for rUTI prevention.
**Molecularly targeted/anti-biofilm therapies**	In vitro and animal studies; small human pharmacokinetic pilot for FimH inhibitor	UPEC and murine UTI models; 6 healthy human volunteers for 1-deoxymannose	Experimental controls/free compounds depending on study	Not applicable; human pilot n = 6	Experimental; human pilot urine collection 1–8 h after administration	Bacterial adhesion/burden or urinary excretion; no clinical rUTI recurrence endpoint	No clinical preventive effect estimate/CI; FimH antagonist reduced bladder bacterial load ~1000-fold in mice	Preclinical/indirect	No demonstrated clinical efficacy; human study assessed pharmacokinetics/excretion only, not treatment or prevention.
**Precision medicine/biomarkers**	Observational and mechanistic studies	Women/patients with UTI/rUTI; experimental biomarker/genetic studies	Healthy/control populations depending on study	Not applicable as a single population	Variable	Biomarkers/genetic factors associated with recurrence risk; not intervention studies	No therapeutic effect estimate/CI	Indirect	Predictive/associational evidence only; clinical utility and therapeutic benefit have not been established.
**D-mannose**	Large randomized, placebo-controlled clinical trial	Women with recurrent UTI	Placebo	598 randomized (303 d-mannose; 295 placebo)	Treatment/follow-up through study endpoint; primary outcome assessed after randomization	≥1 further episode of clinically suspected UTI requiring contact with ambulatory care	RR 0.92 (95% CI 0.80–1.05); 51.0% vs. 55.7%; risk difference −5% (95% CI −13% to 3%)	Direct	No statistically significant benefit; primary endpoint was clinically suspected rather than universally culture-confirmed UTI; adherence and withdrawals must be considered.

## Data Availability

No new data were created or analyzed in this study. Data sharing is not applicable to this article.
